# CTLA-4 Activation of Phosphatidylinositol 3-Kinase (PI 3-K) and Protein Kinase B (PKB/AKT) Sustains T-Cell Anergy without Cell Death

**DOI:** 10.1371/journal.pone.0003842

**Published:** 2008-12-04

**Authors:** Helga Schneider, Elke Valk, Rufina Leung, Christopher E. Rudd

**Affiliations:** 1 Cell Signalling Section, Department of Pathology, University of Cambridge, Cambridge, United Kingdom; 2 Molecular Immunology Section, Division of Investigative Sciences, Faculty of Medicine, Imperial College London, Hammersmith Hospital, London, United Kingdom; 3 Cambridge Institute for Medical Research, Cambridge, United Kingdom; Universität Heidelberg, Germany

## Abstract

The balance of T-cell proliferation, anergy and apoptosis is central to immune function. In this regard, co-receptor CTLA-4 is needed for the induction of anergy and tolerance. One central question concerns the mechanism by which CTLA-4 can induce T-cell non-responsiveness without a concurrent induction of antigen induced cell death (AICD). In this study, we show that CTLA-4 activation of the phosphatidylinositol 3-kinase (PI 3-K) and protein kinase B (PKB/AKT) sustains T-cell anergy without cell death. CTLA-4 ligation induced PI 3K activation as evidenced by the phosphorylation of PKB/AKT that in turn inactivated GSK-3. The level of activation was similar to that observed with CD28. CTLA-4 induced PI 3K and AKT activation also led to phosphorylation of the pro-apoptotic factor BAD as well as the up-regulation of BcL-XL. In keeping with this, CD3/CTLA-4 co-ligation prevented apoptosis under the same conditions where T-cell non-responsiveness was induced. This effect was PI 3K and PKB/AKT dependent since inhibition of these enzymes under conditions of anti-CD3/CTLA-4 co-ligation resulted in cell death. Our findings therefore define a mechanism by which CTLA-4 can induce anergy (and possibly peripheral tolerance) by preventing the induction of cell death.

## Introduction

CD28 and CTLA-4 have opposing effects on T-cell function by providing positive and negative signals, respectively [1]–[3]. Both bind CD80/86, with CTLA-4 exhibiting a preference for CD80 [4]. CTLA-4 negative regulation was shown by antibody ligation [5], and by the development of autoimmune disease in CTLA-4 deficient mice [6], [7]. CD4 positive CTLA-4-/- T-cells are also resistant to anergy induction and tolerance [8]. In this context, CTLA-4 plays a crucial role in autoimmunity and anti-tumor responses [9]. Since anergy often leads to increased apoptosis, one central question concerns how CTLA-4 can anergize and maintain tolerance without inducing T-cell death. This issue could be key in the development of strategies to modulate tumor and transplant tolerance and rejection.

CTLA-4 binds to phosphatidylinositol 3-kinase (PI 3K) [10] as well as phosphatases PP2A and SHP-2 [11]–[13]. This seeming paradox has yet to be reconciled since phosphatases inhibit signaling events, while PI 3K generates D-3 lipids for recruitment of proteins with pleckstrin homology (PH) domains. Phosphatidylinositol 3,4-biphosphate (PIP_2_) recruits PH domain kinase 1 (PDK1) that activates serine/threonine protein kinase B (PKB/AKT) by phosphorylation of Thr-308 and Ser-473 [14]. PKB/AKT in turn phosphorylates the pro-apoptotic protein BAD and pro-survival mediators such as IkB and the FOXO transcription factor as well as the kinase GSK-3 α/β [15]. BAD, a pro-apoptotic member of the BcL-2 protein family, promotes apoptosis through heterodimerization with anti-apoptotic proteins such as BcL-2 and BcL-XL [16]. BAD binds to BcL-XL/BcL-2 and interferes with their function, while BAD phosphorylation on Ser-136 releases BcL-XL to mediate mitochondrial-dependent pro-survival [17].

Receptor mediated induction of anergy without cell death is key to the maintenance of immune function and peripheral tolerance. One central question concerns the signaling mechanism used by CTLA-4 to induce long-term anergy and prevent the induction of cell death. In this study, we show that CTLA-4 activation of PI 3-K and PKB/AKT sustains T-cell anergy without cell death. CD3/CTLA-4 co-ligation rescued cells from apoptosis under the same conditions that induced T-cell anergy, and this occurred in a PI 3K and PKB/AKT dependent manner. Inhibition of the PI 3K-PKB/AKT pathway resulted in cell death without anergy. Overall, our findings provide a novel mechanism to ensure the maintenance of CTLA-4 mediated non-responsiveness and tolerance in the immune system.

## Results and Discussion

**Figure 1:**
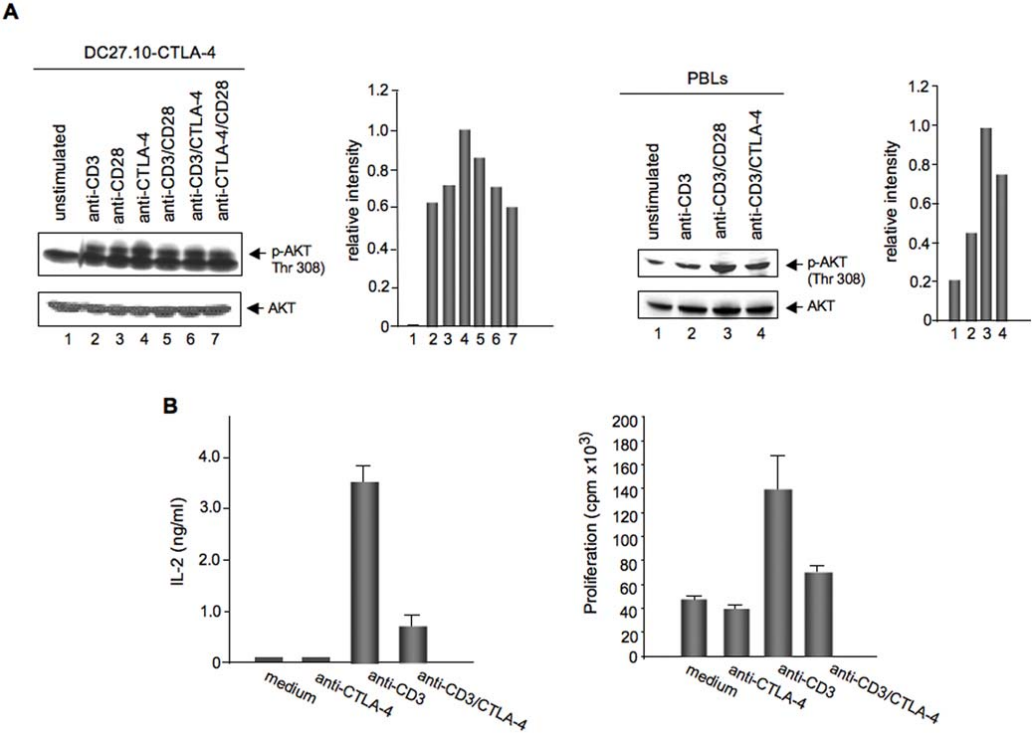
Panel A: CTLA-4 induces phosphorylation of PKB/AKT. Upper left panels: DC27.10-CTLA-4 were either left untreated (lane 1) or stimulated for 30 min with anti-CD3 (lane 2), anti-CD28 (lane 3), anti-CTLA-4 (lane 4), anti-CD3/CD28 (lane 5), anti-CD3/CTLA-4 (lane 6) and anti-CD28/CTLA-4 (lane 7) antibodies. Cell lysates were immunoblotted with anti-phospho-AKT (Thr-308) antibody (lanes 1–7). Histogram depiction of phosphorylated AKT as detected by densitometric reading. Lower panel: Equal amounts of cell lysates were immunoblotted for AKT (lanes 1–7). Upper right panels: Pre-activated T-cells were either left untreated (lane 1) or stimulated for 30 min with anti-CD3 (lane 2), anti-CD3/CD28 (lane 3) and anti-CD3/CTLA-4 (lane 4) antibodies. Cell lysates were immunoblotted with anti-phospho-AKT (Thr-308) (lanes 1–4) antibody. Histogram depiction of phosphorylated AKT as detected by densitometric reading. Lower panel: Equal amounts of cell lysates were immunoblotted for AKT (lanes 1–4). Panel B: CTLA-4 mediated inhibition of IL-2 production and proliferation. DC27.10-CTLA-4 cells and peripheral T-cells were either left unstimulated, or stimulated with anti-CTLA-4, anti-CD3 and anti-CD3/CTLA-4 mAbs. After 24 hours, IL-2 production was measured by ELISA. After 48 hours, proliferation was measured by [^3^H] thymidine incorporation. Bar graphs show mean±SD. Results are representative of at least three experiments.

Given that CTLA-4 can induce non-responsiveness without apoptosis, a key question concerned the underlying mechanism. The maintenance of cell survival during anergy induction is needed for long-term tolerance in transplantation. It was therefore important to investigate whether binding of CTLA-4 to PI 3K leads to activation of PKB/AKT and its downstream targets such as GSK3 α/β and BAD to mediate cell survival. To assess this, pre-activated peripheral CTLA-4 positive T-cells, or a T-cell hybridoma expressing CTLA-4 (DC27.10-CTLA-4) were stimulated with anti-CD3, anti-CD3/CD28 or anti-CD3/CTLA-4 mAbs followed by immunoblotting for phosphorylated PKB/AKT (Thr-308) [14], [18] (Fig. 1A, left and right panels). Based on the crystal structure, this site within the activation loop is crucial to the activation of the kinase [18]. In the T-cell hybridoma, anti-CTLA-4 induced phosphorylation of PKB/AKT relative to unstimulated cells (left panel, lane 4 vs. 1; lower band is non-specific; histogram). The level of phosphorylation was comparable to that induced by anti-CD3 and anti-CD28 (lanes 2 and 3, respectively). Co-ligation of CTLA-4 with anti-CD3 revealed phosphorylation at levels similar to anti-CD3/CD28 (lane 6 vs. 5). As a control, immunoblotting with anti-AKT mAb showed equal levels of protein in the cell lysates (Fig. 1A, lower panel). In peripheral T-cells, anti-CD3/CTLA-4 increased phosphorylation of PKB/AKT when compared to anti-CD3 stimulation (right panel, lane 4 vs 2; histogram). In this case, the phosphorylation was lower than observed for CD3/CD28 (lane 3), most probably due to the fact that CTLA-4 is expressed at lower levels than CD28 and given the fact that there is heterogeneity of CTLA-4 expression in primary T-cells. Importantly, activation of PKB/AKT occurred under conditions where anti-CD3/CTLA-4 inhibited TcR/CD3 mediated IL-2 production and proliferation (Fig. 1B) [5]. These observations indicate that CTLA-4 can activate PKB/AKT as shown by the phosphorylation of Thr-308.

**Figure 2:**
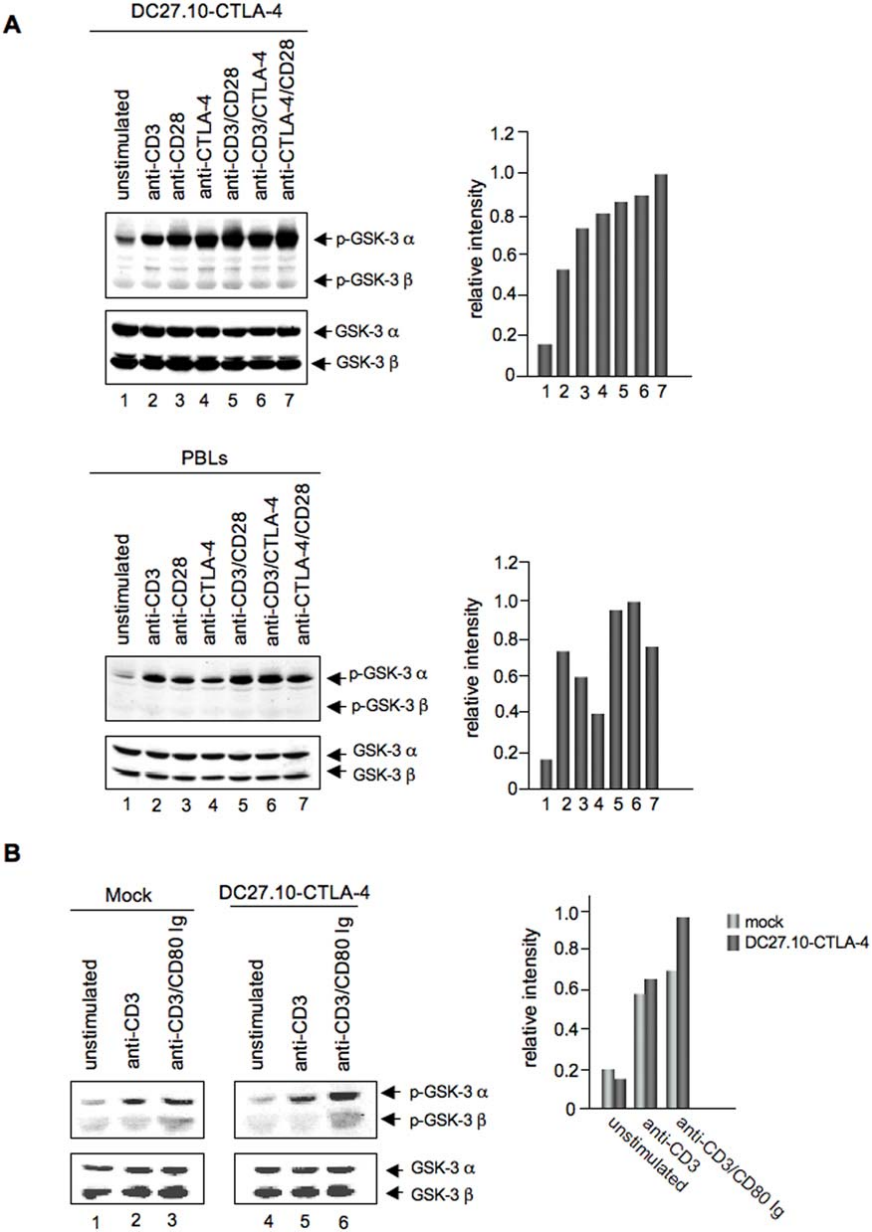
Panel A: CTLA-4 mediated phosphorylation of GSK-3. Upper left panel: DC27.10-CTLA-4 and pre-activated peripheral T-cells were stimulated for 30 min as described for Figure 1A. Cell lysates were immunoblotted with anti-phospho-GSK-3 α/β antibody (lanes 1–7). Upper right panel: Histogram depiction of phosphorylated GSK-3 as detected by densitometric reading. Lower left panel: Cells treated as described above were lysed and immunoblotted with an antibody against total GSK-3 α/β (lanes 1–7). Similar results were obtained from at least three other experiments. Lower right panel: Histogram depiction of phosphorylated GSK-3 as detected by densitometric reading. Panel B: Ligation of CTLA-4 by natural ligand induces phosphorylation of GSK-3. DC27.10 cells transfected with mock (lanes 1–3) or CTLA-4 (lanes 4–6) were either left untreated (lanes 1, 4) or stimulated for 30 min with anti-CD3 (lanes 2, 5) or anti-CD3/CD80Ig (lanes 3, 6) and assesssed for phosphorylation of GSK-3 by immunoblotting with anti-phospho-GSK-3 α/β antibody (lanes 1–6). Lower panel: Equal amounts of cell lysates were immunoblotted for total GSK-3 α/β (lanes 1–6). Right panel: Histogram depiction of phosphorylated GSK-3 as detected by densitometric reading. Results are representative of at least two experiments.

As a read-out for PKB/AKT activity, we next assessed the phosphorylation of the PKB/AKT target GSK-3 α and β on inhibitory serine 21 and 9, respectively [14]. Anti-CTLA-4 readily induced GSK-3 α phosphorylation in DC27.10-CTLA-4 and activated peripheral T-cells (Fig. 2A, left and right panel, lane 4. histograms). The level of phosphorylation was comparable to that induced by anti-CD3 and anti-CD28 (lanes 2 and 3, respectively). Co-ligation of CTLA-4 with CD3 or CD28 often led to increased GSK-3 α phosphorylation (lanes 6, 7). No phosphorylation of the β isoform of GSK-3 could be detected despite equal expression levels of GSK-3 α and β in these cells (lower panel). CTLA-4 mediated phosphorylation of GSK3 α was also observed with natural ligand such as plate-bound CD80Ig together with anti-CD3 (Fig. 2B). Since both CD28 and CTLA-4 are expressed on activated peripheral T-cells (i.e. both bind to CD80), this assay was conducted using the DC27.10 T-cell hybridoma transfected with mock (i.e. express CD28 alone), or with CTLA-4 (i.e. express CD28 and CTLA-4). While anti-CD3 induced comparable GSK-3α phosphorylation in both transfectants (lane 2 vs. 5), CD80Ig/anti-CD3 revealed greater GSK-3 α phosphorylation when CTLA-4 was present (lane 6 vs. 3 and histogram). These observations indicated that CTLA-4 ligation by antibody or natural ligand can activate PKB/AKT and induce GSK-3 α phosphorylation.

**Figure 3:**
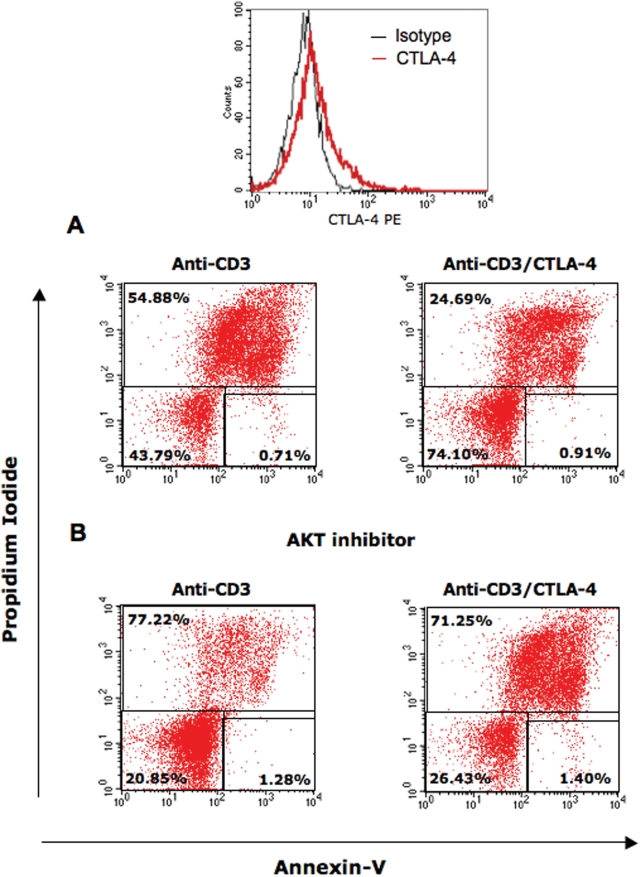
CTLA-4 ligation rescues cells from apoptosis and is dependent on AKT activation. Pre-activated PBLs were stimulated with anti-CD3 (left panel) and anti-CD3/CTLA-4 (right panel) in the absence (upper panel) or presence of AKT inhibitor (AKT inhibitor II) (lower panel). 48 hours later, cells were stained with Annexin V-Cy5 and PI and analysed by FACS for cell death. Top panel shows CTLA-4 surface expression in these cells. Similar results were obtained from at least three other experiments.

Both PKB/AKT and GSK-3 generate pro-survival signals that rescue cells from AICD or apoptosis [19], [20], [21]. Therefore, we first investigated whether CTLA-4 could induce pro-survival signals and secondly, whether these effects would be dependent on PKB/AKT activity. To assess this, peripheral T-cells were pre-activated with anti-CD3 to express surface CTLA-4 (Fig. 3), rested for two days and then re-stimulated with anti-CD3 and anti-CD3/CTLA-4. After 48 hours cells were analysed for cell death. As reported by many labs [22], anti-CD3 induced significant levels of AICD (Fig. 3). About 55 percent of cells underwent AICD in response to CD3 ligation as assessed by AnnexinV/PI staining. However, co-ligation of CTLA-4 increased cell survival to 74 percent versus 44 percent cell survival with anti-CD3 alone (Fig. 3A, upper right panel vs. upper left panel). This finding was also observed at various post-ligation time points (data not shown). Of importance, this rescue from cell death occurred using the same conditions by which CTLA-4 co-ligation inhibited anti-CD3 induced IL-2 production and proliferation (Fig. 1B).

To next assess whether this CTLA-4 induced cell survival was dependent on PKB/AKT activity, cells were cultured in the presence of the AKT inhibitor II. The increase in cell survival that was induced by CTLA-4 was reversed by the addition of the AKT inhibitor (Fig. 3A, lower right panel). In fact, the inhibitor reduced CTLA-4 mediated cell survival to levels comparable to those induced by anti-CD3 (71 percent vs. 77 percent cell death). The inhibition of PKB/AKT activity therefore blocked the induction of the survival signals induced by CTLA-4 co-ligation such that the level of cell survival was similar to anti-CD3. The remaining cells were capable of proliferation as measured by the incorporation of [^3^H]thymidine (data not shown). This observation showed that the ability of CTLA-4 to rescue from cells death occurred in a PKB/AKT dependent manner.

**Figure 4:**
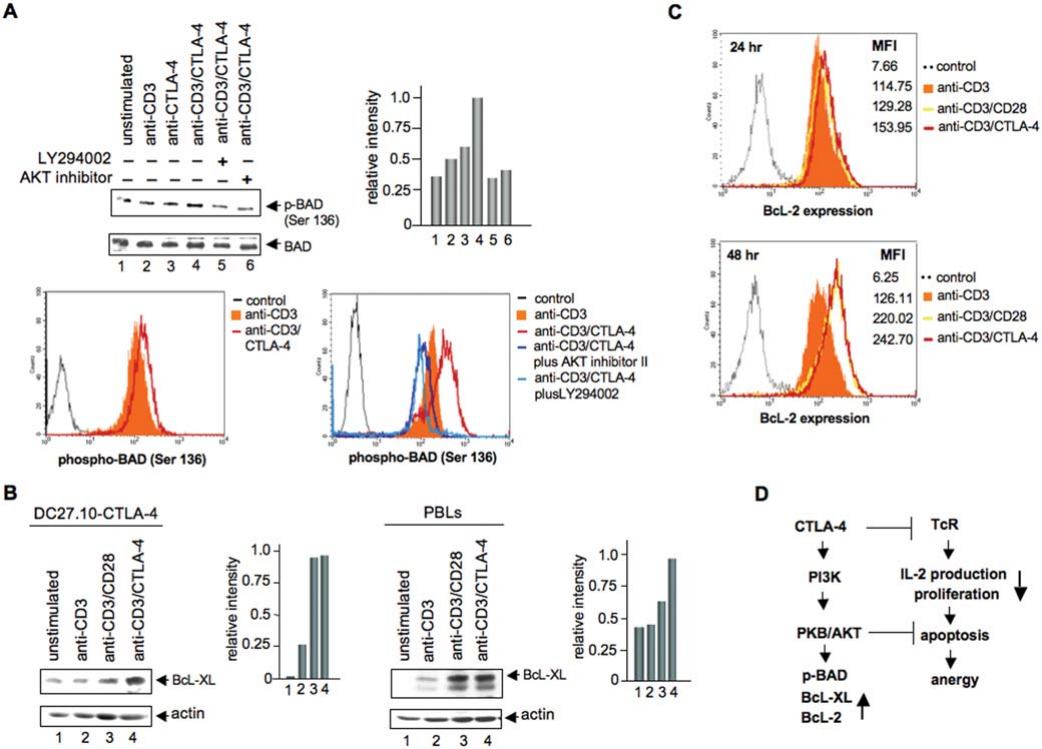
Panel A: CTLA-4 ligation induces phosphorylation of BAD at Ser-136. Upper panel: DC27.10-CTLA-4 cells were either left unstimulated (lane 1) or stimulated for 30 min with anti-CD3 (lane 2), anti-CTLA-4 (lane 3) and anti-CD3/CTLA-4 (lane 4) mAbs. In lane 5 and 6, cells were pretreated with LY 294002 (100 µM, 30 min) or AKT inhibitor II (15 µM, 30 min), respectively and then stimulated with anti-CD3/CTLA-4 antibodies. Cell lysates were immunoblotted with anti-phospho-BAD (Ser-136) antibody (lanes 1–6). Right upper panel: Histogram depiction of phospho-BAD as detected by densitometric reading. Middle panel: Similar amounts of cell lysates were immunoblotted for total BAD (lanes 1–6). Lower panel: Pre-activated PBLs were re-stimulated with anti-CD3 or anti-CD3/CTLA-4 in the absence or presence of AKT inhibitor II or LY 294002. 24 hours later, cells were washed, stained with anti-phospho-BAD (Ser 136)/anti-rabbit AlexaFluo488 antibodies and analysed by flow cytometry. Panel B: CTLA-4 ligation induces up-regulation of BcL-XL. Left panel: DC27.10-CTLA-4 cells were either left unstimulated (lane 1) or stimulated for 24 hours with anti-CD3 (lane 2), anti-CD3/CD28 (lane 3), and anti-CD3/CTLA-4 (lane 4) antibodies. Cell lysates were immunoblotted with anti-BcL-XL antibody (lanes 1–4). Right panel: Pre-activated peripheral T-cells were treated as described above and assessed for BcL-XL expression by immunoblotting with anti-BcL-XL antibody (lanes 1–4). Middle panels: Similar amounts of cell lysates were immunoblotted for actin (lanes 1–4). Panel C: CTLA-4 ligation induces up-regulation of BcL-2. Pre-activated PBLs were re-stimulated with anti-CD3, anti-CD3/CD28 or anti-CD3/CTLA-4 antibodies. 24 and 48 hours later, cells were washed, stained with anti-BcL-2/anti-rabbit AlexaFluo647 antibodies and analysed by flow cytometry. Similar results were obtained from three other experiments. Panel D: CTLA-4 induced pro-survival signaling pathways. CTLA-4 can increase cell survivial under conditions of anti-CD3/CTLA-4 induced non-responsiveness. CTLA-4-PI 3K activates PKB/AKT by phosphorylation at Thr-308 that in turn inactivates pro-apoptotic BAD by phosphorylation at Ser-136. Inhibitors of PI 3K and PKB/AKT blocked this event. Decreased active BAD induced by CTLA-4 ligation was accompanied by increased levels of BcL-XL/BcL-2 expression. BcL-XL/BcL-2 are then able to mediate their mitochondrial-dependent pro-survival effects.

Given the activation of PKB/AKT by CTLA-4, we assessed whether CTLA-4 might rescue anti-CD3 induced AICD by modulating the BAD/BcL-XL pathway. To assess whether this pathway was engaged by CTLA-4, phosphorylation of BAD at Ser-136 was investigated upon CTLA-4 co-ligation. BAD Ser-136 is the major site for BAD phosphorylation by PKB/AKT [23]. As shown in Figure 4A, anti-CTLA-4 increased phosphorylation of BAD at Ser-136 when compared to unligated cells (lane 3 vs. 1; histogram). This phosphorylation level was similar to that mediated by co-ligation with anti-CD3 (lane 4 vs. 3). Anti-CD3 alone had a lower effect than observed for anti-CD3/CTLA-4 (lane 2 vs. 4 and FACS histogram). Inhibition of PI 3K or PKB/AKT resulted in reduced BAD phosphorylation at Ser-136 (lanes 5,6 vs. 4). The total level of BAD expression was monitored by blotting with an anti-BAD antibody (lower panel). These observations indicated that CTLA-4 can activate PKB/AKT in a pathway that phosphorylates and inactivates BAD in its inhibition of BcL-XL and BcL-2 function.

A decrease in BAD function might result in an increase in the expression of BcL-XL. Given this, we next assessed whether anti-CTLA-4 could increase BcL-XL expression as monitored by anti-BcL-XL immunoblotting (Fig. 4B). Up-regulation of BcL-2 by IL-2 has been reported to depend on the PI 3K-PKB/AKT pathway [24]. Further, CD3/CD28 co-ligation leads to an increase in BcL-XL expression [25]. Indeed, anti-CD3/CTLA-4 increased BcL-XL expression relative to anti-CD3 as assessed 24 hours following ligation in both DC27.10-CTLA-4 (left panel, lane 4 vs. 2; histogram) and pre-activated primary T-cells (right panel, lane 4 vs. 2; histogram). The increase was similar or higher than that induced by anti-CD3/CD28 (left and right panels, lane 4 vs. 3) and was PI 3K and PKB/AKT dependent (data not shown). Immunoblotting with an anti-actin antibody showed similar amounts of loaded cell lysates (middle panel). Figure 4C shows the up-regulation of BcL-2 in primary T-cells after 24 and 48 hour stimulation with anti-CD3, anti-CD28 and anti-CTLA-4. These findings indicate that CTLA-4 can increase BcL-XL and BcL-2 expression in a manner similar to CD28, but under conditions that accompany a blockade of anti-CD3 mediated IL-2 production and proliferation (Fig. 1B).

In summary, a central question in the induction of T-cell non-responsiveness concerns the mechanism by which non-responsiveness can be induced without an increase in cell death or apoptosis. Without an ability to protect against cell death, anergy would not be possible due to cell deletion such as in the case of peripheral deletion. In this report, we have uncovered a mechanism by which CTLA-4 can prevent apoptosis without incurring anergy/non-responsiveness (Fig. 4D). We found that the PI 3K-PKB/AKT anti-apoptotic pathway was engaged under the same conditions that led to the classic induction of T-cell non-responsiveness by anti-CD3/CTLA-4 antibody co-ligation. CTLA-4 binds directly to the p85 subunit of PI 3K [26]. While the mechanism by which CTLA-4 induces anergy is still under investigation [3], [11]–[13], it required the PI 3K-PKB/AKT pathway to maintain cell survival under conditions of anergy induction. This was shown by the fact that anti-CD3/CTLA-4 ligation led to extensive apoptosis in the presence of PI 3K and AKT/PKB inhibitors, factors whose phosphorylation and activation was increased by CTLA-4 co-ligation. In another pathway, PP2A, a phosphatase reported to associate with CTLA-4 [13], dephosphorylates BcL-2 and protects this anti-apoptotic protein from proteasome-dependent degradation [28]. The ability of CTLA-4-PI 3K-PKB/AKT to maintain cell survival under conditions of anergy induction would provide a mechanism to ensure long-term tolerance in immunity, as has been observed using CTLA-4 and anti-CD45RB reagents in transplant rejection [29], [30].

Anergy induction is often associated with increased susceptibility to apoptosis [31]. Our findings clearly demonstrated that CTLA-4 ligation can increase survival of cells responding to anti-CD3 (Fig. 3). Reversal of the pro-survival signals by inhibition of PKB/AKT indicates that the PKB/AKT pathway is responsible for this event in a manner similar to CD28 [32]. Interestingly, CTLA-4 induced PKB/AKT activation also led to two events, increased BAD phosphorylation and increased levels of BcL-XL/Bcl-2 expression (Fig. 4). BAD phosphorylation leads to binding and degradation by 14-3-3 proteins and to reduced BAD binding and sequestration of Bcl-XL [33]. CTLA-4 therefore targets BcL-XL by BAD inactivation leading to the increased presence of pro-apoptotic BcL-XL and less degradation. Alternatively, the effects of increasing Bcl-XL expression may be mediated via a different pathway. In either case, CTLA-4 mediates its effects by a combination of providing more active BcL-XL (i.e. less BAD) and greater levels of BcL-XL expression. These findings with primary cells complement previous work by ourselves and others on CTLA-4 effects on Fas-FasL signaling [34], [35]. One prediction from this work is that the modulation of the CTLA-4-PKB/AKT pathway may be exploited to reverse CTLA-4 induced anergy in various physiological conditions such as in the case of tumour rejection. The outcome will depend on the sensitivity of the TcR/CD3 versus CTLA-4 pathways on PKB/AKT signaling. Unlike with CTLA-4, the TcR employs multiple pathways in the activation of PKB/AKT [36]. Future studies will be needed to modulate this CTLA-4 survival pathway for modulating tumor and transplant tolerance and rejection.

## Materials and Methods

### Cells, reagents and antibodies

DC27.10-CTLA-4 cells were cultured in RPMI 1640 medium supplemented with fetal calf serum as described [37]. Peripheral blood lymphocytes (PBLs) were isolated from the buffy coat by centrifugation on a Lymphoprep density gradient. Adherent cells were depleted from the PBLs by plastic adherence after overnight incubation in culture medium. Non-adherent cells were stimulated with anti-CD3 for to 2–3 days, rested for 48 hours and then restimulated with the indicated antibodies (pre-activated PBLs). Anti-phospho-GSK-3 α/β and anti-BAD mAbs were purchased from New England Biolabs (Hertfordshire, UK), anti-GSK-3 α/β and anti-phospho-BAD (Ser-136) mAbs from Biosource (Nivelles, Belgium), anti-AKT1/2 from Autogen Bioclear (Wiltshire, UK), anti-phospho AKT (Thr-308) from New England Biolabs (Hertfordshire, UK), anti-BcL-XL from Cambridge Bioscience (Cambridge, UK), anti-BcL-2 from BD Biosciences (Oxford, UK). Anti-murine CD28 (37.51) was purchased from Pharmingen (Oxford, UK). Anti-murine CD3 (145-2C11) and anti-human CD3 (OKT3) were from American Type Culture Collection. Anti-human CTLA-4 (BNl3) was kindly provided by Dr B. Broeker (Greifswald, Germany). LY294002 was bought from CN Biosciences (Nottingham, UK), AKT inhibitor II from Calbiochem (Nottingham, UK) and recombinant human CD80 Ig from R&D Systems (Abingdon, UK). Annexin V-Cy5 was purchased from BD Pharmingen (Oxford, UK).

### Cell stimulation and immunoblotting

DC27.10-CTLA-4 cells and pre-activated PBLs were stimulated for 30 min with anti-CD3 (2 µg/ml), anti-CD28 (8 µg/ml), anti-CTLA-4 (8 µg/ml), anti-CD3/CD28 (2 µg/ml/8 µg/ml), anti-CD3/CTLA-4 (2 µg/ml/8 µg/ml), anti-CD3/CD80Ig (2 µg/ml/10 µg/ml) and anti-CD28/CTLA-4 (8 µg/ml/8 µg/ml) mAbs together with crosslinking rabbit anti-hamster/mouse antibodies. Immunoblotting was conducted as described [37]. Protein was visualized by enhanced chemiluminescence (ECL; Amersham Pharmacia Biotech).

For BcL-XL protein expression, cells were stimulated with plate-bound anti-CD3 (2 µg/ml), anti-CD3/CD28 (2 µg/ml/8 µg/ml) and anti-CD3/CTLA-4 (2 µg/ml/8 µg/ml) mAbs for 24 and 48 hours. Antibody concentration was adjusted by adding anti-TNP as an isotype-specific mAb.

### Interleukin -2 production and proliferation

For IL-2 production, DC27.10-CTLA-4 cells were stimulated as described [34]. After 24 hours, supernatants were taken and IL-2 production was measured by ELISA (Pharmingen) according to the manufacturer's protocol.

To assess proliferation, peripheral T-cells were either left unstimulated or stimulated with anti-CD3 (2 µg/ml), anti-CTLA-4 (20 µg/ml) or anti-CD3/CTLA-4 (2 µg/ml/20 µg/ml) for 48 hours. 1 µCi of [^3^H] thymidine was added for the last 12 hours of the incubation time. All cell culture groups were done in triplicates.

### Measurement of apoptosis

PBLs were pre-activated with anti-CD3 (5 µg/ml), rested for 48 hrs and re-stimulated for 48 hrs with anti-CD3 (2 µg/ml) or anti-CD3/CTLA-4 (2 µg/ml/20 µg/ml) in the absence or presence of 15 µM AKT inhibitor II. To measure apoptosis, cells were stained after 48 hrs with Annexin V-Cy5 and propidium iodide (PI) according to the manufacturer's protocol. PI was used to identify late apoptotic and dead cells.
