# Correction: CTLA-4 Activation of Phosphatidylinositol 3-Kinase (PI 3-K) and Protein Kinase B (PKB/AKT) Sustains T-Cell Anergy without Cell Death

**DOI:** 10.1371/annotation/5f36e0a6-8e8b-4b33-8e96-a2d53d5c1e46

**Published:** 2009-01-21

**Authors:** Helga Schneider, Elke Valk, Rufina Leung, Christopher E. Rudd

The two histograms in Figure 4B were incorrectly switched. Please view the corrected version of Figure 4 here: 

**Figure 4:**
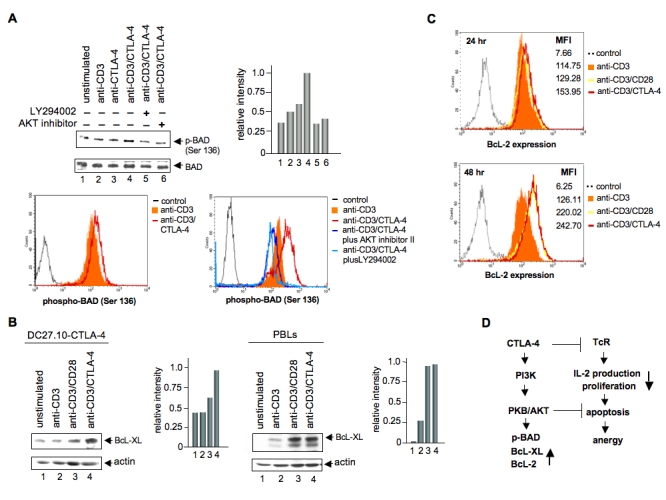
Panel A: CTLA-4 ligation induces phosphorylation of BAD at Ser-136. Upper panel: DC27.10-CTLA-4 cells were either left unstimulated (lane 1) or stimulated for 30 min with anti-CD3 (lane 2), anti-CTLA-4 (lane 3) and anti-CD3/CTLA-4 (lane 4) mAbs. In lane 5 and 6, cells were pretreated with LY 294002 (100 µM, 30 min) or AKT inhibitor II (15 µM, 30 min), respectively and then stimulated with anti-CD3/CTLA-4 antibodies. Cell lysates were immunoblotted with anti-phospho-BAD (Ser-136) antibody (lanes 1–6). Right upper panel: Histogram depiction of phospho-BAD as detected by densitometric reading. Middle panel: Similar amounts of cell lysates were immunoblotted for total BAD (lanes 1–6). Lower panel: Pre-activated PBLs were re-stimulated with anti-CD3 or anti-CD3/CTLA-4 in the absence or presence of AKT inhibitor II or LY 294002. 24 hours later, cells were washed, stained with anti-phospho-BAD (Ser 136)/anti-rabbit AlexaFluo488 antibodies and analysed by flow cytometry. Panel B: CTLA-4 ligation induces up-regulation of BcL-XL. Left panel: DC27.10-CTLA-4 cells were either left unstimulated (lane 1) or stimulated for 24 hours with anti-CD3 (lane 2), anti-CD3/CD28 (lane 3), and anti-CD3/CTLA-4 (lane 4) antibodies. Cell lysates were immunoblotted with anti-BcL-XL antibody (lanes 1–4). Right panel: Pre-activated peripheral T-cells were treated as described above and assessed for BcL-XL expression by immunoblotting with anti-BcL-XL antibody (lanes 1–4). Middle panels: Similar amounts of cell lysates were immunoblotted for actin (lanes 1–4). Panel C: CTLA-4 ligation induces up-regulation of BcL-2. Pre-activated PBLs were re-stimulated with anti-CD3, anti-CD3/CD28 or anti-CD3/CTLA-4 antibodies. 24 and 48 hours later, cells were washed, stained with anti-BcL-2/anti-rabbit AlexaFluo647 antibodies and analysed by flow cytometry. Similar results were obtained from three other experiments. Panel D: CTLA-4 induced pro-survival signaling pathways. CTLA-4 can increase cell survivial under conditions of anti-CD3/CTLA-4 induced non-responsiveness. CTLA-4-PI 3K activates PKB/AKT by phosphorylation at Thr-308 that in turn inactivates pro-apoptotic BAD by phosphorylation at Ser-136. Inhibitors of PI 3K and PKB/AKT blocked this event. Decreased active BAD induced by CTLA-4 ligation was accompanied by increased levels of BcL-XL/BcL-2 expression. BcL-XL/BcL-2 are then able to mediate their mitochondrial-dependent pro-survival effects.

